# Clinical characteristics and treatment outcomes of acute severe autoimmune hepatitis

**DOI:** 10.1186/s12876-021-01653-4

**Published:** 2021-03-01

**Authors:** Linhua Zheng, Yansheng Liu, Yulong Shang, Zheyi Han, Ying Han

**Affiliations:** National Clinical Research Center for Digestive Diseases and Xijing Hospital of Digestive Diseases, Xijing Hospital, Air Force Military Medical University, Xi’an, 710032 China

**Keywords:** Autoimmune hepatitis, Immunoglobulin G, Antinuclear antibody, Centrilobular necrosis, Plasma cell infiltration, Corticosteroid

## Abstract

**Background and aim:**

Acute severe autoimmune hepatitis (AS-AIH) is a rare cause of acute liver failure (ALF), which is often neglected and delayed in treatment. The purpose of this study was to analyze the clinical characteristics and therapeutic effects of AS-AIH.

**Methods:**

Retrospective analysis was performed. AIH was diagnosed according to the International Autoimmune Hepatitis Group (IAIHG) criteria revised in 1999. AS-AIH was defined as an acute presentation (onset of symptoms to presentation of ≤ 26 weeks) and INR of ≥ 1.5, and no histologic evidence of cirrhosis.

**Results:**

Twelve patients were diagnosed as AS-AIH. At baseline, median immunoglobulin G was 28.35 g/L (range, 11.4–49.2). Ten (83.3%) patients were antinuclear antibodies and/or anti-smooth muscle antibodies positive. The prominent histologic characteristics were lobular necrosis/inflammation (91.7%) and plasma cell infiltration (100%). All patients received corticosteroid therapy. Death occurred in 2 (16.7%) patients within 30 days resulted from ALF. The average interval between the onset of symptoms and initiation of corticosteroid therapy in deceased patients was 65 days, compared with 19 days for survivors.

**Conclusions:**

AS-AIH is an uncommon disease with poor outcomes. Patients with acute severe hepatitis of unknown cause should be minded the possibility of AS-AIH and corticosteroids should be considered as soon as possible.

## Introduction

Autoimmune hepatitis (AIH) is an immune-mediated necroinflammatory liver disease. In most of the cases, AIH manifests as a chronic disease. Since it was first reported in 1984, there have been rare but significant cases [1] of acute AIH in recent years [2]. Acute presentation of AIH has been reported, and cases with acute manifestations usually contained two distinct clinical entities [1, 3, 4]. One is true acute AIH without chronic histologic changes (acute form), and the other is acute exacerbation of chronic AIH (acute exacerbation form) [3, 5–10]. The acute form was suggested to be further subdivided into non-severe, severe, and fulminant AIH [11]. It has emerged that acute severe AIH (AS-AIH) is related to a high incidence of acute liver failure (ALF) [11, 12].

Czaja et al*.* and Yeoman et al. previously defined AS-AIH as an acute presentation (onset of symptoms to presentation of ≤ 26 weeks) with INR of ≥ 1.5, and no histologic evidence of cirrhosis [13, 14]. However, patients with AS-AIH often present with atypical symptoms, with normal immunoglobulin G (IgG) and negative serological markers, especially antinuclear antibodies (ANA), anti-liver kidney microsomal 1 (LKM-1) antibody, and anti-smooth muscle antibodies (ASMA) [3, 10, 11, 14–16]. Therefore, liver histopathology, which is crucial to diagnosis of AIH and treatment decision, is considered a prerequisite for its timely detection. Regarding the histopathological features of AS-AIH, centrilobular necrosis with plasma cell infiltration was reported to be more diagnostic than typical AIH features of interface hepatitis and emperipolesis [3, 8, 9]. Particular attention was paid to centrilobular necrosis, which in previous reports has ranged from 21.8 to 100% in patients with acute presentations of AIH [3, 9, 10, 12, 17–19]. However, the significance of centrilobular necrosis remains to be elucidated [11]. Therefore, early diagnosis of AS-AIH remains challenging and urgent.

AIH is the first liver disease demonstrated in clinical trials to be effectively interfered with corticosteroid therapy. However, the efficacy of corticosteroid in AS-AIH is not well verified. In a cohort of 72 acute icteric AIH patients, corticosteroid treatment failure occurred in 18% [20]. It has been reported that the Model for End-Stage Liver Disease (MELD) score was helpful in identifying patients who will probably fail corticosteroid therapy and require liver transplantation [11, 20]. In a single center cohort with similar definition of AS-AIH, higher MELD scores at presentation were associated with treatment failure [21]. In addition, the poor responses to corticosteroid therapy were often attributed to delayed initiation of treatment, particularly as a result of delayed diagnosis [1].

To better acquire the clinical features and an overview of treatment outcomes of AS-AIH, we conducted a retrospective study of patients with AS-AIH in our hospital.

## Methods

All patients with acute presentation of AIH from 2008 to 2018 admitted to Xijing Hospital of Digestive Diseases (Xi’an, Shaanxi, China) was retrospectively analyzed. All patients were treatment naïve. The diagnosis of AIH was based on the International Autoimmune Hepatitis Group (IAIHG) diagnostic criteria revised in 1999. Definite AIH (score ≥ 16) or probable AIH (score ≥ 10) were based on clinical, serological, laboratory, and histological data. We chose the IAIHG diagnostic criteria because it is more comprehensive and sensitive in acute presentation of AIH [22]. Patients with other causes of liver disease including viral hepatitis, alcoholic liver disease, nonalcoholic fatty liver disease, celiac disease, drug induced liver injury, acute biliary diseases, and hereditary or metabolic disorders were excluded.

Due to the absence of the standardized definition, AS-AIH was defined in the present study as an acute presentation (onset of symptoms to presentation of ≤ 26 weeks) and INR of ≥ 1.5, without histologic evidence of cirrhosis [11]. According to the criteria above, of 41 treated-naïve patients totally included with acute presentation of AIH, 29 patients (70.7%) with underlying cirrhosis were excluded from the study.

The samples of liver biopsy were available and independently assessed by two blinded professional liver pathologists based on two criterias [9]: (1) lobular changes including lobular necrosis/inflammation (including spotty, bridging, massive, and centrilobular necrosis), lobular plasma cell infiltration, hepatic rosette, and emperipolesis; (2) portal/periportal changes including interface hepatitis, portal plasma cell infiltration, bile duct injury or fibrosis.

All patients with AS-AIH in our hospital were firstly treated with prednisolone of 40–60 mg/day, which gradually tapered after remission. The common maintenance dose of prednisolone was 5–10 mg/day. MELD score was calculated at the time of admission. Patients with high MELD score of ≥ 18 on admission or significant deterioration of liver function within 3 days after corticosteroids therapy were assessed for liver transplantation. If the patients refused to list for liver transplantation after they were fully informed the potential benefits and risks of liver transplantation, essential therapy would still be provided and the patients should be strictly and dynamically evaluated.

### Statistical analysis

The various liver test parameters were analyzed using the SPSS V24.0 (Chicago, IL).

## Results

### Characteristics of AS-AIH patients at admission

A total of 12 treated-naïve patients (11 females and 1 male, age ranges: 36–68 years) were identified with AS-AIH. All patients met the 1999 revised IAIHG criteria for a definite (6, 50%) or probable (6, 50%) diagnosis of AIH. The most common symptoms were fatigue, jaundice, and anorexia in all patients. Other symptoms included diarrhea, vomiting, itching, and shortness of breath. No definite clinical manifestation of encephalopathy was found in these patients at the time of treatment initiation. The clinical characteristics of all patients at admission were summarized in Table 1. At baseline, median alanine aminotransferase (ALT), alkaline phosphatase (ALP), total bilirubin (TBIL), international normalized ratio (INR), and MELD scores were 277.5 IU/L (range, 165–1223), 178.0 IU/L (range, 73–336), 295.25 μmol/L (range, 175.40–533.10), 1.72 (range, 1.53–1.92), and 23.5 (range, 22–27), respectively. Median serum IgG level in these patients was 28.35 g/L (range, 11.40–49.20). A more than 1.1 times of the upper limit of normal (ULN) of IgG was found in 8 (66.7%) patients. And 7 (58.3%) patients were only ANA-positive (≥ 1:100), 1 (8.3%) only ASMA-positive, and 2 (16.7%) both ANA-positive and ASMA-positive. No patients were positive for anti-LKM-1.

**Table 1:** Clinical, biological and immunological characteristic at admission of the 12 patients with AS-AIH

Patients	Clinical and biological parameters						Immunological			
	Age (year)/gender(M/F)	ALT (IU/L)	ALP (IU/L)	TBIL (μmol/L)	INR	MELD score	IgG (g/L)	ANA	ASMA	Anti-LKM-1
1	50/F	205	124	252.8	1.58	25	18.5	1:320	n	n
2	45/F	267	73	337.4	1.88	25	49.2	1:320	p	n
3	68/M	165	159	242	1.63	22	31.9	n	n	n
4	53/F	279	237	268.7	1.53	22	12.2	n	p	n
5	55/F	408	336	321.8	1.57	23	44.9	1:160	n	n
6	58/F	311	130	175.4	1.89	22	28.5	n	n	n
7	54/F	228	211	216.1	1.92	23	23.4	1:320	p	n
8	43/F	1223	197	329.6	1.86	27	35.6	1:320	n	n
9	55/F	498	216	250.3	1.57	22	28.2	1:5120	n	n
10	36/F	276	153	378.5	1.68	24	16.1	1:3200	n	n
11	54/F	214	245	461	1.75	25	11.4	1:320	n	n
12	65/F	313	101	533.1	1.89	27	28.5	1:100	n	n

ALT, alanine aminotransferase; ALP, alkaline phosphatase; TBIL, total bilirubin; INR, international normalized ratio; MELD, the model for end stage liver disease scores; IgG, immunoglobulin G; ANA, antinuclear antibodies; ASMA, anti-smooth muscle antibodies; anti-LKM-1, anti-liver kidney microsomal-1 antibody

p, positive; n, negative

### Histologic evaluation

The histologic characteristics of all patients at admission were summarized in Table 2. Among the 12 patients with AS-AIH, 11 (91.7%) patients showed lobular necrosis/inflammation (spotty 2, bridging 2, massive 1, and centrilobular necrosis 6), and 12 (100%) patients showed plasma cell infiltration in lobular or portal areas (lobular areas 8, lobular and portal areas 4). Interface hepatitis was present in only 3 (25%) patients, with mild to moderate presentation. Hepatic rosette was observed in only 2 (16.7%) patients. Mild to moderate bile duct injury was observed in 4 (33.3%) patients. No patients showed fibrosis.

**Table 2:** Histological features of the 12 patients with AS-AIH

Patients	Plasma cell infiltration	Lobular necrosis/inflammation	Interface hepatitis	Hepatic rosette	Bile duct injury	Fibrosis
1	PC1	L1	a	a	a	0
2	PC1	L1	a	a	a	0
3	PC1	L2	a	a	a	0
4	PC1	L2	a	a	p	0
5	PC1,2	L3	p	a	p	0
6	PC1	a	a	a	a	0
7	PC1	L1	a	a	a	0
8	PC1	L1	a	a	a	0
9	PC1,2	L1	p	p	p	0
10	PC1,2	L3	p	a	a	0
11	PC1,2	L1	a	p	p	0
12	PC1	L4	a	a	a	0

PC1, lobular area; PC2, portal area

L1, centrilobular necrosis; L2 spotty necrosis; L3 bridging necrosis; L4 massive necrosis

a, absent; p, present

Fibrosis was assessed with the Batts-Ludwig scoring system (F0-4)

### Outcomes of the patients

Among the 12 patients with AS-AIH, 2 (16.7%) died at 15 and 20 days after corticosteroid therapy as consequences of ALF with mild encephalopathy. In those 2 cases, the intervals between onset of symptoms and initiation of corticosteroid therapy were about 60 and 70 days, respectively. After 3 days of corticosteroid therapy, changes of the 2 patients were seen in TBIL levels from 461 to 493 μmol/L, 533.1 to 600 μmol/L. These 2 patients with significant deterioration of the clinical status decided to list for liver transplantation as we suggested, and corticosteroid therapy was discontinued at day 3. However, they did not undertake liver transplantation owing to mismatch. After 3 days of initiation of corticosteroid therapy, the remaining survivors had improved clinical status and liver function variables. The maintenance dose of prednisolone was 5–10 mg/day during their follow-up. The changes of liver function parameters at day 3 posttreatment were presented in Table 3. The remaining survivors had a median follow-up period of 52.5 months (range: 24–117 months). In addition, 1 patient experienced disease relapse and progressed to liver cirrhosis after 1 year of follow-up. This patient was given prednisolone of 40 mg/day plus azathioprine of 50 mg/day. And prednisolone was gradually tapered after remission and azathioprine was maintained for treatment. But no autoantibody-negative AS-AIH patients evolved to -positive in long-term follow-up. No adverse event due to the corticosteroid treatment was reported.

**Table 3:** Outcomes of the 12 patients with AS-AIH treated with corticosteroids

Patients	Days before treatment (days)	Changes (Δ) of Day-3 postcorticosteroid treatment			Alive (Y/N)	Relapse (Y/N)	Follow-up period (months)
		Δ ALT (IU/L)	Δ TBIL (μmol/L)	Δ INR			
1	20	−65	−41	−0.08	Y	N	75
2	7	−102	−10.1	−0.25	Y	N	117
3	10	−47	−45	−0.13	Y	N	108
4	15	−21	−12.8	−0.01	Y	N	25
5	30	−201	−37	+ 0.02	Y	N	61
6	20	−146	−60.4	−0.29	Y	N	29
7	40	−15	−2.5	−0.5	Y	N	44
8	10	−382	−40.3	−0.34	Y	N	24
9	8	−78	−2.3	+ 0.03	Y	N	66
10	30	−34	−21.6	+ 0.02	Y	Y	28
11	60	−12	+ 32	+ 0.25	N		
12	70	+ 37	+ 66.9	+ 0.01	N		

Δ, change; Y, yes; N, no; −, decrease; + , increase

Days before treatment: intervals between onset of symptoms and initiation of corticosteroid therapy

## Discussion

The clinical features of AS-AIH in our cohort as follows: (1) the most common symptoms were fatigue, jaundice, and anorexia; (2) afflicted patients showed elevated levels of ALT and TBIL, while no patients showed fibrosis, which was compatible with inclusion criteria for AS-AIH in previous study [11]; (3) a quarter of patients exhibited normal IgG levels, and a quarter showed ANA negative; (4) the most prominent histologic characteristics were lobular necrosis/inflammation and plasma cell infiltration in lobular and/or portal areas, which in line with the characteristics reported in earlier studies [3, 8, 9]; (5) the timing of treatment initiation and the changes of liver function (mainly TBIL level) at day 3 posttreatment may be associated with the outcomes of corticosteroid therapy.

Due to the high TBIL levels and an INR of ≥ 1.5, acute severe hepatitis can be diagnosed easily in clinical work. In AS-AIH, however, patients may be autoantibody negativity and with normal IgG, and individual histological features are less specified. Therefore, there has been no standardized definition or golden diagnostic criteria for AS-AIH. It was reported by Yeoman et al. that 88% of AS-AIH patients tested positive for ANA, ASMA, or anti-LKM-1 [14]. And ANA antibodies were undetected or weakly positive in about 29%-39% of patients with fulminant AIH [10, 15]. In fulminant AIH or AS-AIH, gamma-globulin levels could be normal in 25%–39% patients [10, 11, 15]. A Japanese nationwide survey indicated that IgG levels in more than half of patients with acute presentation of AIH were below the ULN [23]. It has been hypothesized that severe AIH with underlying liver failure may induce a state of immune dysfunction, which may mask the antibody formation early in some of the patients [24]. It suggests that sequential testing later in the disease course might result in late emergence of conventional antibodies. This was shown in a study of AIH in children where 56% had acute presentation and conventional autoantibodies were absent in 17.3% [25]. They subsequently became positive for ANA, ASMA, and/or anti-LKM-1 on follow-up. In our cohorts, 10/12 patients (83.3%) were ANA-positive and/or ASMA-positive, 8/12 patients (66.7%) displayed serum IgG higher than 1.1 times ULN. But no autoantibody-negative patients evolved to -positive in long-term follow-up. The Japanese nationwide survey revealed that ANA-positive and -negative AIH patients with acute presentation found no significant differences in clinical status, histopathological findings or outcomes [23]. Serologically negativity seemed to be a characteristic of severe AIH. Therefore, the absence of conventional autoantibodies or normal IgG levels, which may delay the diagnosis and influence the outcomes, cannot exclude the diagnosis of AS-AIH.

Liver biopsy is essential to support the diagnosis of AIH and to exclude differential diagnosis. The classical histologic features of chronic AIH include interface hepatitis, portal inflammation, or hepatocyte rosettes. In contrast with classic AIH, the histological diagnosis of AS-AIH is difficult with absence of specific features and overlap with viral hepatitis and drug induced liver injury [11]. In the acute hepatitis phase, patients usually exhibit histological features of acute hepatitis, such as centrilobular inflammation/necrosis, without or with minimal periportal hepatitis, such as interface hepatitis. Actually, it was reported that centrilobular necrosis could be observed in most cases of acute presentation of AIH [3, 9, 10, 12, 17–19]. And many studies supported that centrilobular necrosis might be a histologic characteristic of AS-AIH. However, some studies also found that centrilobular necrosis was a marker of severity in both early and chronic AIH [11, 18]. Interestingly, previous studies reported that some patients with acute presentation of AIH showed lobular hepatitis without the involvement of centrilobular or portal areas or demonstrated lobular hepatitis with slight portal inflammation without centrilobular necrosis [9]. The reason for this difference is not yet clear, but several possible explanations may be proposed, such as different diagnostic criteria, different disease severities, and different definition of centrilobular necrosis. The findings may reflect disease severity and indicate new acute-onset disease. Plasma cell infiltration is not specific, but still is the most characteristic feature of AIH. It has been reported that plasma cell enrichment was noted in 77% patients with AS-AIH in a cohort [14]. In our cohorts, the most prominent features were lobular necrosis/inflammation (including centrilobular necrosis) and plasma cell infiltration in lobular or portal areas. Interface hepatitis, hepatic rosette, and bile duct injury were also observed in some patients. It suggests that acute AIH has extensive histological changes in lobular, centrilobular and portal areas.

AIH is the first liver disease demonstrated in clinical trials to be effectively interfered with corticosteroid therapy. However, it was reported that AS-AIH being less effective than chronic AIH treated with corticosteroids [26]. Ichai et al. suggested that using corticosteroids in severe or fulminant AIH had no benefit. This conclusion, however, came from 16 atypical patients with deficiency of IAIHG scores and hepatic histology, all of whom had coagulation disorders, and 10 of whom with hepatic encephalopathy [27]. Of other 72 treatment-naïve AIH patients (with jaundice but without encephalopathy), 18% experienced corticosteroid failure, defined as requiring second-line treatment, and progression to liver failure or death [20]. Yeoman et al. included a well-characterized cohort of 32 patients of similar definition of AS-AIH, which showed a significantly higher number of patients undergoing liver transplantation in the untreated group as compared with those receiving corticosteroids [14]. Mortality was 19% in their group of AS-AIH as compared with 16.7% in our study. Benefit from early institution of corticosteroid therapy has been shown in 36% to 100% of patients with severe AIH in various retrospective and small studies [12, 26, 28]. Zachou et al. performed an open, real-world observational study and found that prompt initiation of high-dose corticosteroids in original AS-AIH seemed safe and efficient [29]. And for the patients with ALF induced by AIH, corticosteroids was also found to improve their outcome [30]. On the data of our patients with well-characterized AS-AIH, we suggest that the timing of treatment initiation and the changes of liver function at day 3 posttreatment may be associated with the outcomes of corticosteroid therapy, as was seen in 2 of our patients with AS-AIH who died as consequences of ALF. Previous studies showed that the presence of encephalopathy, TBIL level, INR, platelet counts, MELD score and the improvement of bilirubin on day 7 of treatment were the predictors of corticosteroid response [11, 20, 21]. There are also studies that showed 2 weeks after the introduction of corticosteroid was a critical point [31], and an increase of bilirubin or INR during the first 2 weeks should lead to liver transplantation or second-line treatment [32]. But it is still controversy that when to continue or stop corticosteroids for liver transplantation. However, on multivariable analysis, neither the histologic feature nor the presence or absence of serological markers was a predictor of treatment response [23]. These findings indicated that the prognosis of patients was associated with the severity of the initial disease, and that corticosteroid treatment should therefore be initiated as early as possible before the disease progresses and worsens. Although a proportion of patients with AS-AIH respond to corticosteroids, for the patients with ALF, transplantation remains the best option. Patients with a higher incidence of ALF should be considered for liver transplantation. It is critical to identify those with a higher likelihood of nonresponding to corticosteroids. Overall, one of 10 remaining survivors (10%) occurred disease relapse in our study. Previous studies showed that disease relapse could be observed in 30% of AIH patients with acute presentation and 24% with chronic phase [23, 33], indicating that disease relapse should be considered in both acute and chronic phase of AIH.

There are several limitations in the present study. It was a retrospective study of a small number of patients, but all the cases were both clinically and histologically confirmed. Indeed, autoantibody and IgG are required both in revised IAIHG scoring and the simplified AIH diagnosing criteria. When we only relied on the scoring systems, we may underestimate the number of AS-AIH. It should be noted that there are no standard diagnostic criteria for AS-AIH till now, and further studies need to be performed to better define this special group of AIH patients.

In conclusion, AS-AIH is a newly discovered atypical disease. The diagnostic hallmarks of AS-AIH, such as IgG, ANA, and histologic features, are needed to fully evaluated. However, physicians must consider the possibility of AS-AIH when dealing with patients with acute liver dysfunction of unknown cause, as delayed diagnosis and treatment can lead to a poor prognosis of AS-AIH.

## Data Availability

The datasets used and/or analysed during the current study available from the corresponding author on reasonable request.

## Abbreviations

AS-AIH: Acute severe autoimmune hepatitis
ALF: Acute liver failure
IAIHG: International Autoimmune Hepatitis Group
AIH: Autoimmune hepatitis
IgG: Immunoglobulin G
ANA: Antinuclear antibodies
LKM-1: Liver kidney microsomal 1
ASMA: Anti-smooth muscle antibodies
MELD: Model for End-Stage Liver Disease
INR: International normalized ratio
ALT: Alanine aminotransferase
ALP: Alkaline phosphatase
TBIL: Total bilirubin
ULN: Upper limit of normal
